# Clinico-radiological features of optic nerve sheath schwannoma: Review and illustrative case

**DOI:** 10.1177/11206721241287575

**Published:** 2024-09-28

**Authors:** Clare Quigley, Jessica Y Tong, Alexander S Zhang, Alkis J Psaltis, Dinesh Selva

**Affiliations:** 1 South Australian Institute of Ophthalmology, 1062 Royal Adelaide Hospital, Adelaide, Australia; 2 Department of Surgery - Otolaryngology, Head and Neck Surgery, 1066 The University of Adelaide, Basil Hetzel Institute for Translational Research, Woodville South, Adelaide, Australia

**Keywords:** Orbital surgery < ORBITAL DISEASE, eyelid disease < oculoplastic eyelid /lacrimal disease, tumors/Neoplasms < orbital disease, diagnostic studies < orbital disease, tumors / neoplasms < tumors

## Abstract

The optic nerve sheath is a rare site for schwannoma, to our knowledge 19 optic nerve sheath schwannoma (ONSS) cases have been reported. Difficulty can arise in diagnosis as imaging findings can be relatively non-specific. We describe a case of ONSS that mimicked orbital cavernous venous malformation pre-operatively. A 43-year-old woman presented with right subacute visual loss, reduced vision to 6/48 and signs of optic neuropathy. Endocapsular excision of the mass, which was adherent to the optic nerve, was accomplished from an endoscopic endonasal approach utilizing a 5-hand technique of retrocaruncular dissection. Histology showed spindle-shaped tumour cells with S100 positivity, consistent with ONSS. At 6 months post-operatively vision had improved to 6/6. We show that an endoscopic endonasal approach, augmented by transcaruncular retraction, can be utilized to excise ONSS with an excellent outcome. We review published cases of ONSS, including demographic and clinical features, differential diagnosis based on radiological features, and described outcomes, which are generally poor.

## Introduction

Schwannoma is classified as a nerve sheath tumour, arising from the myelin-producing Schwann cells. The nerve of origin is most commonly sensory, with vestibular schwannoma, or acoustic neuroma, the most common tumour site.^1^ Schwannoma is associated with neurofibromatosis type 2 (NF2), an autosomal dominant syndrome caused by mutation in the *NF2* suppressor gene located at chromosome 22q11.2, in 2–18% of cases.^2–4^ The *NF2* gene encodes merlin protein, regulating cell growth, particularly in Schwann cells, and cell-cell adhesion.^5^

Orbital schwannoma is uncommon, and generally arises from sensory branches of the trigeminal nerve.^4,6^ Optic nerve sheath schwannoma (ONSS) is rare, and unusual because of an absence of Schwann cells in association with the optic nerve. ONSS as a distinct entity has been questioned; Kashkouli et al. have described it as a misnomer.^7^ To our knowledge 19 cases of ONSS have been described previously in 17 reports.^7–20^ Some cases were not consistent with ONSS, as the tumour was found easily separable from the optic nerve, or the tumour was a recurrence after surgery at another centre, raising doubt as to the initial primary site and correct classification.^9^ We consider it likely that ONSS arises from sympathetic nerves lying within the optic nerve sheath, innervating the ophthalmic artery and its branches,^21^ and another putative source of ONSS is from ectopic Schwann cells. The originating nerve of ONSS may not be definitively identified.

We describe an ONSS case that demonstrated non-specific imaging findings pre-operatively. Written informed consent for this case report was given by the patient. We review the range of clinical and imaging features, the management and the outcomes described of ONSS in the literature. For the purpose of description of median visual acuity (VA), LogMAR (Logarithm of the Minimal Angle of Resolution) acuity of 1.8, 2.4, 2.7, and 3 were assigned for VA of counting fingers (CF), hand motions (HM), light perception (LP) and non- light perception (NLP) respectively.^22^

## Case description

**Figure 1.**
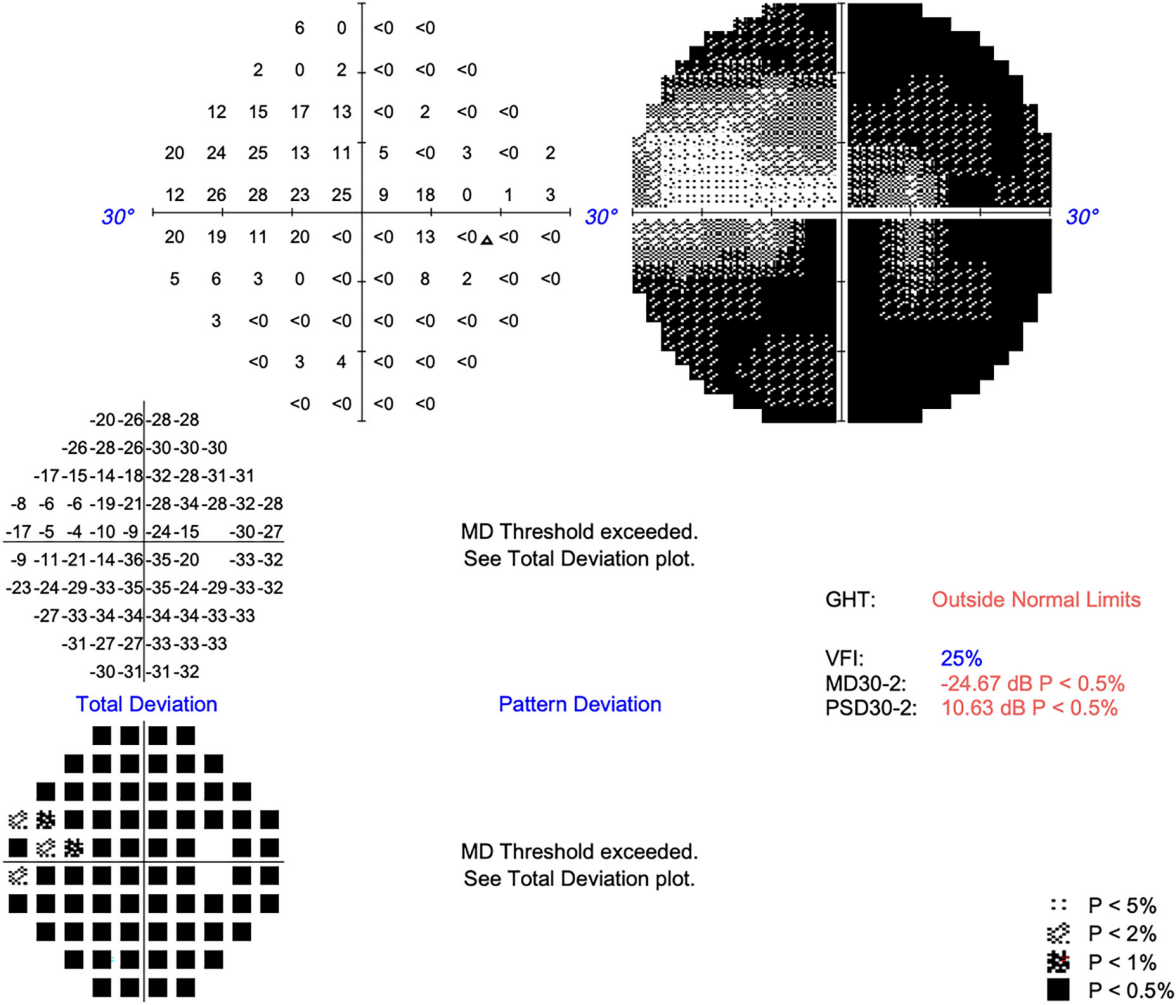
Severely reduced visual field at baseline (mean deviation −24.67 dB, Humphrey 30-2).

**Figure 2.**
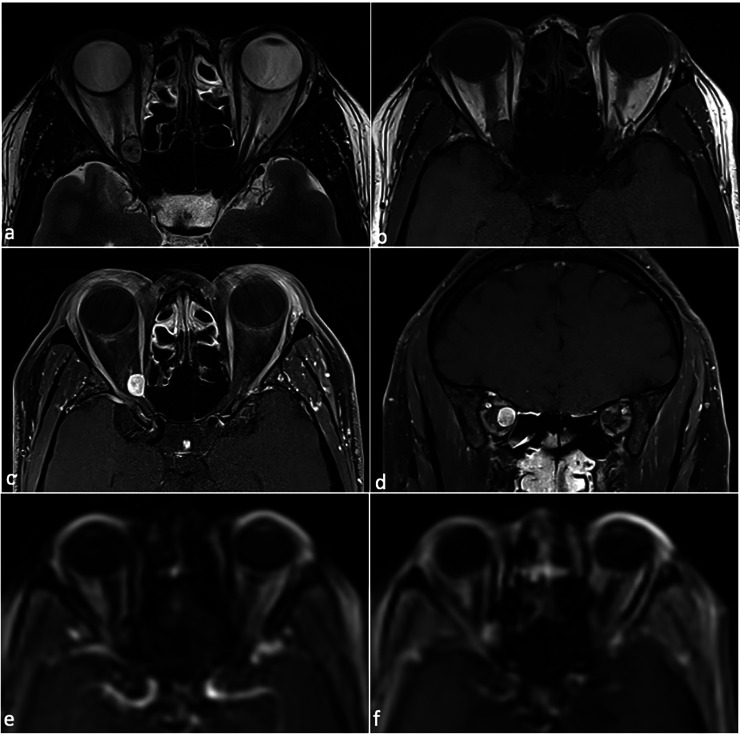
MRI findings. Images showing a well-circumscribed ovoid intraconal mass at the apex of the right orbit, medial to optic nerve, that is T2 isointense (a), isointense on T1 (b), and shows contrast enhancement on axial (c) and coronal (d) T1, T1 dynamic contrast enhancement (DCE) images show focal enhancement that progressed to fill the mass (e-f). Intra-operatively the mass was firmly adherent to the optic nerve and histology was consistent with an optic nerve sheath schwannoma.

A 43 year-old woman was seen in the eye clinic with a two month history of right sided vision loss. Background medical history included chronic rhinosinusitis, obesity and asthma, and medications included Phentermine, Symbicort inhaler, and Mirena coil. There was no personal or family history of neurofibromatosis. On examination she had reduced right VA of 6/120 unaided, pin-holing to 6/60, reduced colour vision reading the Ishihara test plate only, grossly reduced Humphrey visual field with mean deviation (MD) of −24.67 dB (Figure 1), and a positive right relative afferent pupillary defect (RAPD). There was no proptosis. Left VA and optic nerve function were normal. There was mild limitation of right eye abduction. Magnetic resonance imaging (MRI) revealed a well-circumscribed ovoid orbital mass in the intraconal space at the apex of the right orbit, of 9.5 mm maximal linear dimension, adjacent to the optic nerve. It was T1 isointense, T2 hyperintense, and enhanced diffusely with contrast (Figure 2). The MRI dynamic contrast enhanced sequence appeared to demonstrate focal enhancement in the lateral component of the mass followed by gradual and diffuse filling of the remainder of the mass. In combination, these radiological features were suggestive of an orbital cavernous venous malformation, with schwannoma and solitary fibrous tumour considered as differentials. Mucosal thickening suggestive of anterior ethmoid sinusitis was also seen on MRI. Because of the radiological findings of sinusitis, a short course of oral antibiotics and steroids was given pre-operatively.

A joint procedure was performed by the Oculoplastic and Rhinology teams under general anaesthetic, with the assistance of image guidance. Septoplasty was performed through a left hemitransfixion incision, and a septal window was fashioned. Right uncinectomy, antrostomy, anterior and posterior ethmoidectomy, sphenoidectomy and draf IIa frontal clearance was performed, without any finding of pus in the sinuses. The lamina was skeletonised, drilling of the orbital process of the palatine bone and lesser wing of the sphenoid was performed, and a section of the lamina papyracea/medial orbital wall was removed to expose the periobita. The medial periorbita was incised to expose the orbital fat. Prolapsing extraconal and intraconal fat were excised carefully with a laryngeal skimmer blade. An attempt was made to visualise the lesion by endonasal retraction of the medial and inferior recti, but the mass could not be identified. A decision was made to provide retraction via an external approach. Following a medial conjunctival peritomy, the medial rectus was identified and disinserted on 6-0 vicryl. After a retrocaruncular dissection the medial and inferior recti were retracted via a combined external and endonasal 5-hand technique.

**Figure 3.**
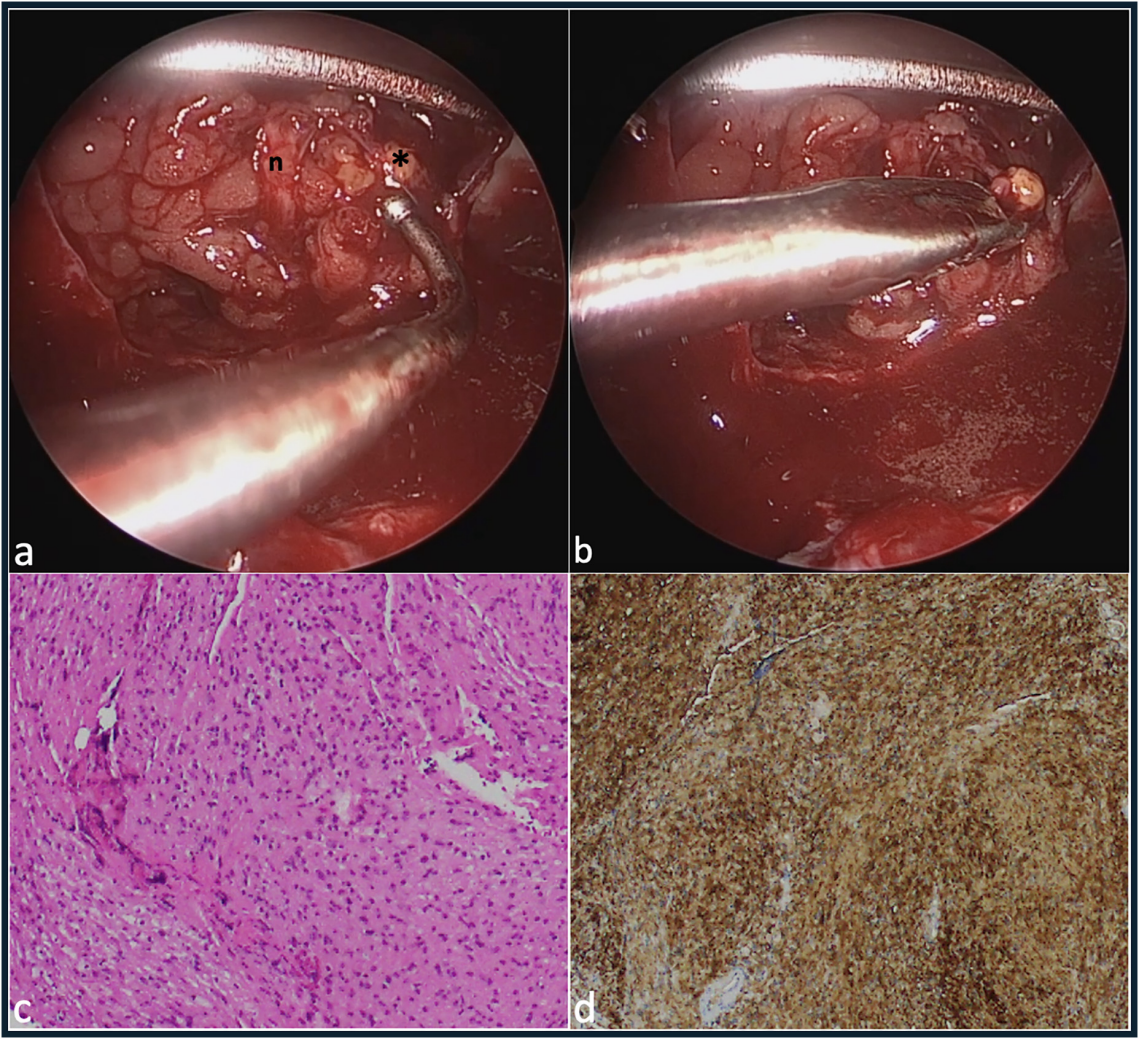
Intra-operative endoscopic view and post-operative histology. With the peri-orbita opened, following orbital fat excision and medial rectus detachment, and with the assistance of transcaruncular dissection and retraction, the oculomotor nerve branch to the medial rectus is visible running vertically (n), anterior to the optic sheath schwannoma (*), which is teased away from surrounding tissue at the apex with an antrum ball probe (a), and is endocapsular excision is performed with a mizuho micro-spoon forceps (b), hematoxylin and eosin staining showed a spindle cell lesion with bland nuclear features, eosinophilic cytoplasm and focal palisading of nuclei (c), positivity for S100 in the tumour cells was consistent with schwannoma (d).

The apical orbital lesion was eventually identified, and teased away from surrounding tissue with an antrum ball probe. The mass, which was white and had a fibrous texture, appeared to have reduced in dimension by approximately 50–60% compared to what it had measured on preoperative imaging, presumably due to the pre-operative course of oral prednisolone that was prescribed to treat her rhinosinusitis. There was a firm attachment to the optic nerve sheath, with traction on the lesion resulting in movement of the globe. The mass was not associated with the divisions of the oculomotor nerve, and the branch to the medial rectus was separately identified anterior to the lesion in the intraconal space. Opening of the capsule lead to extrusion of yellow material. A subtotal endocapsular resection was performed with a mizuho micro-spoon forceps (video, Figure 3(a) and (b)). Histology was sent. Haemostasis was performed and the conjunctiva and caruncle were closed with 8–0 vicryl. Closure of the hemitransfixion incision was performed with 4-0 vicryl rapide.

Histological analysis showed a spindle cell lesion with bland nuclear features, eosinophilic cytoplasm and focal palisading of nuclei. The cells demonstrated focal areas of nuclear enlargement. Mitotic activity was not seen. Staining was positive for S100 in the tumour cells, consistent with schwannoma (Figure 3(c) and (d)).

**Figure 4.**
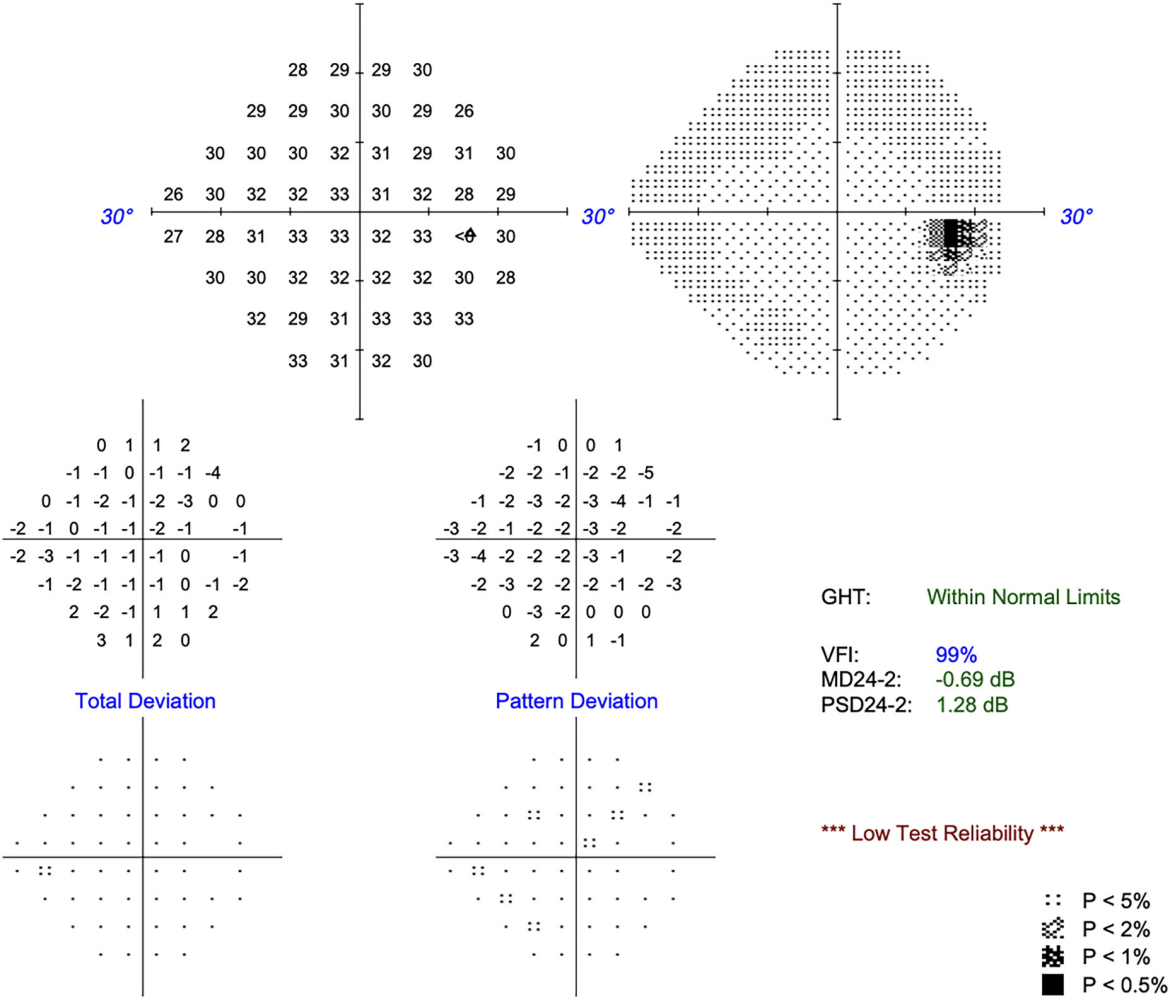
Visual field at 6 months post-op, normal (mean deviation −0.69 dB, 24-2).

At day 1 she had total visual recovery with VA 6/6, but also had a complete ptosis with absent levator function, −4 adduction, infraduction and −2 supraduction. At six months post-operatively VA was 6/6, visual field testing was normal (Figure 4), ptosis had resolved with normal levator function of 18 mm, and ocular improvements improved to normal.

## Discussion and review

### Clinical presentation

Nerve sheath tumours account for less than 5% of orbital masses.^23^ ONSS is very rare, making diagnosis challenging, especially as some of its radiological features are shared with other well-circumscribed orbital tumours, including orbital cavernous venous malformation (OCVM), solitary fibrous tumours and well-circumscribed lymphoma.^24^ ONSS appears to occur in the 4^th^ decade with men and women showing similar incidence, compared to OCVM which is more common in women.^25,26^ Presenting features of ONSS are relatively non-specific compared to other orbital tumours, including proptosis and vision loss most commonly,^12,27^ followed by pain,^16^ and diplopia, commonly gradually progressing over months to years. Visual acuity is typically reduced to blindness at presentation and remains poor post-operatively, see Table 1.^12^

**Table 1.** Clinico-radiological features of optic nerve sheath schwannoma.

No	Article	Age in years	Sex	Presentation	Imaging	Surgery	Outcome
1	Kulkarni et al, 1980	15	M	Gradual loss of vision, proptosis	Not performed	Lateral orbitotomy and tumour excision	Preservation of globe for cosmesis
2	Simpson et al, 1987	11	M	Gradual loss of vision VA: LP	MRI: tortuous optic nerve	Frontotemporal craniotomy and tumour excision	Improvement in vision VA: CF at 3 weeks
3	Saini et al, 1990	45	F	Gradual loss of vision, proptosis VA: LP	CT: well-circumscribed cystic mass with patchy enhancement	Medial orbitotomy and tumour excision	Total loss of vision, no recurrence at 2 years VA: NLP
4	Kim et al, 2002^a^	10	F	Visual field defect VA: 6/9	MRI: ovoid enhancing optic foramen mass	Craniotomy	Visual field improved to normal
5		20	M	Original presentation: reduced vision and ataxia	MRI: Recurrence of tumour, huge supracellar cystic mass	Craniotomy	further recurrence 1 year later: bifrontal craniotomy. 1 year post this surgery: RVA 6/18, LVA 6/60
6	Leruez et al, 2013	65	M	Gradual loss of vision VA: 6/9	MRI: ovoid well-circumscribed intraconal mass enhancing heterogeneously	Lateral orbitotomy and tumour excision	Stable visual acuity, normal visual field post-op
7	Ramey et al, 2015	46	M	Gradual loss of vision	MRI: small ovoid homogeneously enhancing mass medial to optic nerve	Endoscopic endonasal approach: optic nerve decompression and subtotal tumour resection External beam radiotherapy post-op	Stable vision at 3 months
8	Barhmi et al, 2016	57	F	Gradual loss of vision, severe proptosis VA: NLP	MRI: Large heterogeneous intraconal mass filling orbital space	anterior orbitotomy, tumour excision and exenteration	No recurrence at 2 years
9	Junaid et al, 2018	16	F	Proptosis x 3 years VA: NLP	MRI: Large orbital mass with intracranial extension	pterional craniotomy	No recurrence at two years VA NLP
10	Mahore et al, 2019	22	M	Loss of vision over 2 months, proptosis VA: NLP	MRI: Large ovoid intraconal mass, T1: isointense, T2 : hyperintense, significant contrast enhancement	frontal craniotomy and orbitotomy : subtotal excision	Improvement of vision at 2 months VA 6/12
11		39	M	Loss of vision, diplopia, proptosis x 4 months VA 6/60	MRI : Large round intraconal mass, T1 : isointense, T2 : hyperintense, significant contrast enhancement	Lateral orbitotomy	Improvement of vision and diplopia post-operatively VA 6/12
12	Golden, 2019	26	F	Gradual loss of vision, proptosis VA: NLP	CT: Large ovoid intraconal mass filling most of orbit extending to cavernous sinus through superior orbital fissure	Frontotemporal craniotomy and orbital osteotomy, tumour excision	Ptosis and ophthalmoplegia that resolved at 3 months
13	Kashkouli et al, 2019	22	M	Gradual loss of vision, proptosis, VA: 6/18, post EBRT: VA LP	MRI: heterogeneous cystic lesion in intraconal space	Initial biopsy followed by EBRT, progression at 3 months, proceeded to Lateral orbitotomy	Visual loss (NLP), no recurrence at 5 years
14	Xiao et al, 2020	60	F	Gradual loss of vision, headache VA: 6/18	MRI: well-circumscribed cystic mass at cistern	Craniotomy	Improved vision at 1 year VA 6/12
15	Kashkouli et al, 2019	52	M	Visual loss VA LP	MRI: well-circumscribed round apical intraconal mass	Combined medial and lateral wall orbitotomy	NLP, no recurrence at 11 years
16	Sharma et al, 2023	29	F	Proptosis VA: 6/9	MRI: well-circumscribed ovoid heterogeneous intraconal mass	Frontal craniotomy and orbitotomy	Stable vision and improved proptosis at 3 months
17	Benzalim et al, 2023	48	F	Gradual loss of vision, pain, proptosis VA: NLP	MRI: cystic large intraconal mass	Orbitotomy and partial excision	Improvement in proptosis, no improvement in vision post-op
18	Vishwakarma et al, 2024	68	M	Loss of vision x 3 months VA: NLP	MRI: well-defined ovoid mass in medial intraoconal space	Endoscopic endonasal excision	no recurrence at one year VA: NLP
19	Lune et al, 2024	34	F	Proptosis for 1 year, VA 6/6	MRI: well-defined ovoid mass in intraconal space	Lateral orbitotomy	Stable vision post-operatively VA 6/6
20	Quigley et al, 2024	43	F	Gradual loss of vision VA: 6/60	MRI: well-circumscribed ovoid mass in posterior intraconal space	Endoscopic endonasal decompression and transcaruncular orbitotomy with subtotal excision	Improvement in vision post-op with improving ophthalmoplegia VA 6/6 at 6 months
Summary		41 years (11-68) median age (range)	n= 9 M n= 9 F	LP (6/6- NLP) median VA (range)			CF (6/6-NLP) median VA (range)

Sex: male (M), female (F). Imaging: computed tomography (CT), magnetic resonance imaging (MRI), visual acuity (VA), counting fingers (CF), hand motions (HM), light perception (LP), non- light perception (NLP).

^a^ excluded from summary statistics as case descriptions not in keeping with not optic nerve sheath schwannoma.

### Radiological findings

Imaging characteristics of ONSS can also mimic other entities. In our case as in other ONSS described,^9,12,15,19,20^ MRI findings of a well-circumscribed T1 isointense, T2 hyperintense avidly enhancing intraconal ovoid mass were initially suggestive of OCVM.^8,23^ However the T2 heterogeneity seen on MRI favoured schwannoma.^28^ Alternate ONSS MRI findings may include a large mass >2 cm in maximal linear dimension,^8,13,16^ that may include intracranial extension,^18^ or cystic lesions. A cystic structure may arise due to integration of mucinous or microcystic regions, hyaline degeneration, and insufficient vascular supply leading to focal necrosis and haemorrhage, followed by resorption.^8,29^ An MRI appearance of schwannoma as optic nerve thickening has also been described.^10^ Schwannoma enhancement with contrast varies, described as heterogeneous, homogeneous, or avid.^8,12,13,15–17,23^

MRI supplemented with dynamic contrast enhancement (DCE) is useful in differentiating orbital masses.^30^ Schwannoma enhancement typically follows a more heterogeneous pattern than OCVM.^31^ In orbital schwannoma the initial enhancement may start towards the margin of the mass from a larger origin,^32^ as in our case where enhancement was seen initially towards the lateral side of the tumour. Time-signal intensity curves generated from MRI-DCE studies may be used to differentiate benign from malignant orbital lesions, and in the future, they may be further refined to differentiate the radiologically similar OCVM and schwannoma lesions.^30^

### Treatment considerations

Orbital schwannoma may arise from various nerves in the orbit. Firm adhesion to the optic nerve distinguishes ONSS from schwannoma originating from other nerves. This characteristic will not be detectable on pre-operative imaging and becomes apparent intra-operatively when traction on the mass produces visible movement of the globe, as occurred in our case. Upon identification of this sign, further traction on the mass should be minimised to avoid excess stretch on the optic nerve. Miyamura et al. described two cases of intra-optic-canal schwannoma but intra-operatively there was easy separation of the schwannoma from the optic nerve, indicating that these were unlikely to be ONSS.^33^

Evidence of pre-operative ethmoid sinusitis prompted pre-operative treatment with oral antibiotics and steroids, which likely had multiple effects. During surgery there was no pus in the sinuses, a beneficial effect. Shrinkage of the tumour was another likely effect of the steroid pre-treatment. This may have explained the initial difficulty experienced in identifying the mass intraoperatively, which appeared to have reduced in size by 50–60% of the dimensions demonstrated on pre-operative imaging. This necessitated opening the conjunctiva to provide transcaruncular retraction to locate the tumour. A clinical response to steroid of vestibular schwannomas has been described, whereby hearing improvement occurs in patients treated pre-operatively with oral prednisolone.^28^

Described surgical approach to ONSS involve endocapsular excision, as for orbital-cranial schwannoma,^28^ which relieves compression on the optic nerve and generates a histology sample to confirm the diagnosis, while reducing the risk of worsening visual loss. Preservation of vision precludes total extracapsular excision of the tumour. In our case there was rapid and significant improvement in vision post-operatively. The initial postoperative CN3 palsy had also resolved at 6 months follow-up.

Endonasal approaches to orbital biopsy have evolved recently. Access to the medial wall of the orbit and retraction of tissues can be facilitated by 3-hand technique, involving fashioning a septal window to give access to a second surgeon in a binostril approach. In our case, a transcaruncular dissection and retraction technique combined with a binostril approach enabled a 5-hand technique to expose the lesion.^34^ Structures impinging on visualisation of an apical intraconal lesion from an endonasal approach can include the medial rectus, and orbital fat. Medial rectus detachment is safe and was required in our case.^35^ Orbital fat may be carefully excised,^36^ which was performed in our case using a laryngeal skimmer blade. Upon exposure of the lesion, specialised endoscopic instrumentation is required to perform excision. This was performed with the mizuho micro-spoon forceps for this case.

In other similar cases, an endonasal approach has been taken for excision. Takahashi et al. also employed a combined transcaruncular and endoscopic endonasal approach to a series of orbital apical lesions, including a schwannoma that was adherent to surrounding tissues, though optic nerve adherence was not reported.^37^ Ramey et al. reported a joint approach by neurosurgery and rhinology teams utilizing a more posterior approach in the optic canal. This necessitated opening of the dura, with repair of the dura following subtotal excision of the ONSS, without CSF leak.^17^ In a case reported by Vishwakarma et al., where total visual loss had already occurred, an endoscopic endonasal approach was undertaken to ONSS excision.^20^ Endonasal approach may vary with experience and expertise, we find that access via the medial wall, with the mentioned supportive techniques, allows adequate lesion visualisation and excision.

In our case, as in most cases of ONSS published, patients did not undergo radiotherapy, though this has been described in two cases*.*^7,17^ Ramey et al. treated with external beam radiotherapy (EBRT) post-operatively after worsening of visual field testing and radiological evidence of persistent tumour.^17^ Following diagnostic biopsy, Kashkouli et al. treated with EBRT, but after initial improvement there was loss of vision and tumour enlargement prompting tumour excision from a lateral orbitotomy approach.^7^ In general, the longer term outcomes for these patients are not well known, in terms of risk of potential recurrence, with 2 year follow-up without recurrence the latest described time point.^13,23^ Longer reporting timeframes would be beneficial. Final visual outcome shows substantial variation, but is generally poor, with the median post-operative visual acuity count fingers in the cases we identified on review of the literature. While the existence of ONSS as a distinct entity has been challenged,^7^ it may be useful to consider schwannomas which are firmly adherent to the optic nerve as a distinct group, that can present difficulties in management and generally carry a poor prognosis. A limitation of our case description is our short duration of follow-up (6 months), we plan surveillance every 6 months.

## Conclusions

In conclusion, optic nerve sheath schwannoma is a rare tumour that can provide a diagnostic dilemma and may mimic other more common orbital masses, including orbital cavernous venous malformation. Histological sampling is essential, and excision can be accomplished with an excellent visual outcome from an endoscopic endonasal approach.

## Supplemental Material

sj-crdownload-1-ejo-10.1177_11206721241287575 - Supplemental material for Clinico-radiological features of optic nerve sheath schwannoma: Review and illustrative case Supplemental material, sj-crdownload-1-ejo-10.1177_11206721241287575 for Clinico-radiological features of optic nerve sheath schwannoma: Review and illustrative case by Clare Quigley, Jessica Y Tong, Alexander S Zhang and Alkis J Psaltis, Dinesh Selva in European Journal of Ophthalmology

## References

[1] Russell DS Rubinstein LJ . Pathology of tumours of the nervous system. 5th ed. London: Edward Arnold, 1989.

[2] Ferner RE . The neurofibromatoses. Pract Neurol 2010; 10: 82–93. 20308235 10.1136/jnnp.2010.206532

[3] Schatz H . Benign orbital neurilemoma. Sarcomatous transformation in von Recklinghausen's disease. Arch Ophthalmol 1971; 86: 268–273. 4999435 10.1001/archopht.1971.01000010270006

[4] Shields JA . Diagnosis and management of orbital tumours. Philadelphia: Saunders, 1989.

[5] Amaravathi A Oblinger JL Welling DB , et al. Neurofibromatosis: molecular pathogenesis and natural compounds as potential treatments. Front Oncol 2021; 11: 698192. [published Online First: 20210917]. 34604034 10.3389/fonc.2021.698192 PMC8485038

[6] Rose GE Wright JE . Isolated peripheral nerve sheath tumours of the orbit. Eye (Lond) 1991; 5: 668–673. 1800164 10.1038/eye.1991.123

[7] Kashkouli MB Abdolalizadeh P Jafari S , et al. Is primary optic nerve sheath schwannoma a misnomer? Report of two cases and literature review. Orbit 2019; 38: 419–423. [published Online First: 20181116]. 30444169 10.1080/01676830.2018.1545239

[8] Mahore A Ramdasi R Chagla A , et al. Intraconal optic sheath schwannoma: report of two cases. Br J Neurosurg 2019; 33: 101–103. [published Online First: 20170310]. 28281374 10.1080/02688697.2017.1297768

[9] Kim DS Choi JU Yang KH , et al. Optic sheath schwannomas: report of two cases. Childs Nerv Syst 2002; 18: 684–689. [published Online First: 20021016]. 12483351 10.1007/s00381-002-0663-3

[10] Simpson RK Jr. Harper RL Kirkpatrick JB , et al. Schwannoma of the optic sheath. J Clin Neuroophthalmol 1987; 7: 219–222. 2963027 10.3109/01658108709007455

[11] Kulkarni RG Satalkar VY Bhawthankar AW . Neurilemmoma of optic nerve. Indian J Ophthalmol 1980; 28: 95–96. 7216356

[12] Sharma A Singh D Saran R . Primary optic nerve sheath schwannoma: a case report. Br J Neurosurg 2023; 37: 1333–1335. [published Online First: 20210108]. 33416410 10.1080/02688697.2020.1869181

[13] Saini JS Mohan K Sharma A . Primary orbital optic nerve sheath schwannoma. Orbit 1990; 9: 97–99.

[14] Golden N . Optic nerve sheath schwannoma of the orbit: a case report. Bali Med J 2019; 8: 179–182. [published Online First: 20181231].

[15] Leruez S Gohier P Menei P , et al. [Optic nerve schwannoma]. J Fr Ophtalmol 2013; 36: e49–e53. [published Online First: 20130123]. 10.1016/j.jfo.2012.01.014 23352709

[16] Benzalim M Ondima H Alj S . Primary optic nerve sheath schwannoma: a case report and literature review. Radiol Case Rep 2023; 18: 4211–4213. [published Online First: 20230918]. 37745769 10.1016/j.radcr.2023.08.085 PMC10514387

[17] Ramey WL Arnold SJ Chiu A , et al. A rare case of optic nerve schwannoma: case report and review of the literature. Cureus 2015; 7: e265. [published Online First: 20150418]. 10.7759/cureus.265 PMC4494577 26180689

[18] Junaid M Bukhari SS Rashid MU . Optic nerve schwannoma: neurofibromatosus type-1? A case report. J Pak Med Assoc 2018; 68: 950–952. 30323367

[19] Lune A Magdum R Pokle S , et al. Optic Nerve Schwannoma: A Report of a Rare Case From India and Literature Review. Cureus 2024; 16. doi: 10.7759/cureus.59824. [published Online First: May 07, 2024]. PMC11156426 38846181

[20] Vishwakarma R Maheshwari J Soni H . Endoscopic Excision of Optic Nerve Schwannoma. Laryngoscope 2024. doi: 10.1002/lary.31248. [published Online First: 20240106]. 38183323

[21] Francois P Lescanne E Velut S . The dural sheath of the optic nerve: descriptive anatomy and surgical applications. Adv Tech Stand Neurosurg 2011; 36: 187–198. doi: 10.1007/978-3-7091-0179-7_7 21197611

[22] Schulze-Bonsel K Feltgen N Burau H , et al. Visual acuities “hand motion” and “counting fingers” can be quantified with the freiburg visual acuity test. Invest Ophthalmol Vis Sci 2006; 47: 1236–1240. 16505064 10.1167/iovs.05-0981

[23] Barhmi I Mahdoufi R Khallouq A , et al. Uncommon presentation of orbital schwanomma: a case report. Int J Surg Case Rep 2016; 26: 173–175. [published Online First: 20160728]. 27497041 10.1016/j.ijscr.2016.07.045 PMC4976612

[24] Roelofs KA Juniat V O'Rouke M , et al. Radiologic Features of Well-circumscribed Orbital Tumors With Histopathologic Correlation: A Multi-center Study. Ophthalmic Plast Reconstr Surg 2024. doi: 10.1097/IOP.0000000000002584. [published Online First: 20240112]. 38215460

[25] Vahdani K Rose GE . Presenting characteristics for symptomatic, as compared to asymptomatic (assumed). Orbital Cavernous Venous Malformations. Ophthalmic Plast Reconstr Surg 2022; 38: 546–550. [published Online First: 20220503]. 35502799 10.1097/IOP.0000000000002195

[26] Yan J Wu Z . Cavernous hemangioma of the orbit: analysis of 214 cases. Orbit 2004; 23: 33–40. 15513018 10.1076/orbi.23.1.33.28992

[27] Hemalatha AL Vani D Giripunja M , et al. Rare retro-orbital intraconal occurrence of benign schwannoma - a case report. J Clin Diagn Res 2013; 7: 2964–2965. [published Online First: 20131215]. 24551692 10.7860/JCDR/2013/6827.3810 PMC3919384

[28] Huynh PP Saba ES Hoerter JE , et al. Steroid efficacy on audiologic recovery in patients with sudden sensorineural hearing loss and vestibular schwannoma: a retrospective review. Otol Neurotol 2023; 44: 780–785. [published Online First: 20230718]. 37464465 10.1097/MAO.0000000000003954

[29] Gunduz K Shields CL Gunalp I , et al. Orbital schwannoma: correlation of magnetic resonance imaging and pathologic findings. Graefes Arch Clin Exp Ophthalmol 2003; 241: 593–597. [published Online First: 20030618]. 12819974 10.1007/s00417-003-0681-1

[30] Jittapiromsak N Hou P Liu HL , et al. Dynamic contrast-enhanced MRI of orbital and anterior visual pathway lesions. Magn Reson Imaging 2018; 51: 44–50.[published Online First: 20180427]. 29709464 10.1016/j.mri.2018.04.016

[31] Xian J Zhang Z Wang Z , et al. Evaluation of MR imaging findings differentiating cavernous haemangiomas from schwannomas in the orbit. Eur Radiol 2010; 20: 2221–2228. 20393718 10.1007/s00330-010-1774-y PMC2914262

[32] Tanaka A Mihara F Yoshiura T , et al. Differentiation of cavernous hemangioma from schwannoma of the orbit: a dynamic MRI study. AJR Am J Roentgenol 2004; 183: 1799–1804. 15547232 10.2214/ajr.183.6.01831799

[33] Miyamura S Yamaguchi S Takeda M , et al. Pure intra-optic canal schwannoma: report of two cases. Asian J Neurosurg 2017; 12: 797–800. 29114316 10.4103/1793-5482.185071 PMC5652128

[34] Curragh DS Halliday L Selva D . Endonasal approach to orbital pathology. Ophthalmic Plast Reconstr Surg 2018; 34: 422–427. 30059393 10.1097/IOP.0000000000001180

[35] Wu W Selva D Jiang F , et al. Endoscopic transethmoidal approach with or without medial rectus detachment for orbital apical cavernous hemangiomas. Am J Ophthalmol 2013; 156: 593–599. [published Online First: 20130628]. 23810472 10.1016/j.ajo.2013.05.001

[36] Curragh DS Selva D . Endoscopic orbital fat decompression for the management of proptosis in grave's orbitopathy using a laryngeal skimmer blade. Eye (Lond) 2019; 33: 1924–1929. [published Online First: 20190708]. 31285569 10.1038/s41433-019-0519-7 PMC7002766

[37] Takahashi Y Nishimura K Yo K , et al. Resection of orbital apex tumours in the medial orbit via four-handed endonasal and transcaruncular approaches. Eur J Ophthalmol 2024; 34: 864–869. [published Online First: 20230920]. 37731331 10.1177/11206721231204189

